# Authors’ reply to the comment from Glass et al.

**DOI:** 10.1186/s13054-023-04599-z

**Published:** 2023-08-29

**Authors:** Yuki Kotani, Alessandro Pruna, Alessandro Belletti, Todd C. Lee, Giovanni Landoni

**Affiliations:** 1 Department of Anesthesia and Intensive Care, IRCCS San Raffaele Scientific Institute, Via Olgettina 60, 20132 Milan, Italy; 2 School of Medicine, Vita-Salute San Raffaele University, Via Olgettina 58, 20132 Milan, Italy; 3 Department of Intensive Care Medicine, Kameda Medical Center, 929 Higashi-cho, Kamogawa, Chiba 296-8602 Japan; 4 Division of Infectious Diseases, Department of Medicine, McGill University, Montreal, QC Canada

Our meta-analysis suggested a 10% mortality increase when using propofol in critical care and perioperative settings [1], provoking worldwide discussion and attracting multiple letters-to-the-editor. Subsequently, the Editor-in-Chief confirmed the scientific integrity of our paper [2]. In this latest letter, we want to address three points that Glass et al. made.

First, our data extraction strategy, detailed in another reply [3], was appropriately applied to the Likhvantsev et al. study. Nonetheless, when restricting analyses to the evaluable population, a substantial probability of mortality increase (99.1%) remains in the cardiovascular setting (Additional file 1: Table S1).

Second, we confirm the correct exclusion of our large MYRIAD randomized controlled trial (RCT) with patients receiving either total intravenous anesthesia (TIVA) or ≥ 30 min of a volatile agent [4]. Since our meta-analysis [1] pooled studies randomizing patients to propofol versus any comparator, there was no way to correctly include MYRIAD. The choice of intravenous agent was not randomized but left to the practitioner and 23% of TIVA group did not receive propofol. Within the volatile arm, those who received a volatile agent may have received hours of a combination of other agents. Indeed, propofol was used in 22% of cases. Thus, any comparison of those who received propofol with those who didn’t was not randomized within this RCT. Unpublished 1-year mortality supports a 10% mortality increase, consistent with our meta-analysis (2.6% [50/2027] in patients randomized to the volatile group and not receiving propofol as maintenance versus 3.0% [84/2838] in patients who received propofol irrespective of randomized allocation). Notably, we kept strict inclusion criteria also with another large RCT [5] suggesting a propofol detrimental effect on survival persisting until 1 year. We did not include this study in our meta-analysis either, since not meeting our prespecified strict inclusion/exclusion criteria.

Finally, we would like to comment on the concept of spin. All published work has a central thesis and the degree to which one agrees/disagrees with that thesis determines how much readers feel the message has been spun. Whether or not one agrees with the message of our meta-analysis, the data imply a substantial probability of increased mortality with propofol. It is up to the scientific community, profession societies, and individual clinicians to determine their comfort in continuing the status quo. As the Editor-in-Chief wrote [2], our meta-analysis adds to the overall evidence, it is not a final word on the safety of propofol.

### Supplementary Information

**Additional file 1**. Supplemental Table 1.

## Data Availability

Further information on the original manuscript is available from the corresponding authors upon reasonable request.

## Abbreviations

MYRIADL: Mortality in cardiac surgery randomized controlled trial of volatile anesthetics
RCT: Randomized controlled trial
