# Supplementary material for: Authors’ reply to the comment from Glass et al
Source: Crit Care. 2023 Aug 29;27:334. doi: 10.1186/s13054-023-04599-z (PMC10464268; doi:10.1186/s13054-023-04599-z)
Supplement: Supplementary file 1 — Additional file 1. Supplemental Table 1. [file 13054_2023_4599_MOESM1_ESM.docx]

Supplemental Table 1. Additional analyses for the cardiovascular setting with different data extraction strategy for Likhvantsev et al study [1]

| Data extraction strategy | Risk ratio (95% confidence interval) | Probability of risk ratio >1.0 |
| --- | --- | --- |
| Original manuscript | 1.46 (1.13–1.89) | 99.8% |
| Evaluable population of Likhvantsev et al. study^a^ | 1.36 (1.06–1.76) | 99.1% |

^a^ 81 deaths out of 326 in the propofol arm vs. 52 deaths out of 292 in the comparator arm.

Supplemental reference

1. Likhvantsev VV, Landoni G, Levikov DI, Grebenchikov OA, Skripkin YV, Cherpakov RA. Sevoflurane Versus Total Intravenous Anesthesia for Isolated Coronary Artery Bypass Surgery With Cardiopulmonary Bypass: A Randomized Trial. J Cardiothorac Vasc Anesth. 2016;30:1221–7.
